# Feasibility and Safety of Transthoracic Echocardiography-Guided Transcatheter Closure of Atrial Septal Defects with Deficient Superior-Anterior Rims

**DOI:** 10.1371/journal.pone.0051117

**Published:** 2012-12-17

**Authors:** Gui-Shuang Li, Hai-De Li, Jie Yang, Wen-Quan Zhang, Zong-Shen Hou, Qing-Chen Li, Yun Zhang

**Affiliations:** 1 The Key Laboratory of Cardiovascular Remodeling and Function Research, Chinese Ministry of Education and Chinese Ministry of Health, Department of Cardiology, Qilu Hospital Shandong University, Jinan, Shandong, China; 2 Yishui People's Hospital, Yishui, Shandong, China; 3 Department of Pediatrics, Qilu Hospital Shandong University, Jinan, Shandong, China; 4 Dezhou People's Hospital, Dezhou, Shandong, China; University of Virginia Health System, United States of America

## Abstract

Although previous studies showed that transthoracic echocardiography (TTE) can be used to guide transcatheter closure of atrial septal defect (ASD), whether TTE can be used to guide transcatheter closure of secundum ASD with a deficient superior-anterior rim is unknown and this critical issue was addressed in the present study. A total of 280 patients with secundum ASD who underwent transcatheter ASD closure were recruited and divided into groups A and B depending on ASD superior-anterior rim>4 mm (n = 118) or ≤4 mm (n = 162). TTE was used to guide Amplatzer-type septal occluder (ASO) positioning and assess residual shunt. Procedure success was defined as no, trivial and small residual shunt immediately after the procedure as assessed by color Doppler flow imaging. Group A and group B did not differ in complication rate (8.55% vs.7.55%), procedure success rate (98.3% vs. 95.0%) or complete closure rate immediately after the procedure (89.7% vs. 89.3%) or at 6-month follow-up (98.3% vs. 96.8%). The mean procedure and fluoroscopy time in group B were much longer than those in group A. In conclusion, the absence of a sufficient superior-anterior rim in patients undergoing percutaneous closure of secundum-type ASDs using fluoroscopic and TTE guidance is associated with slightly greater device malposition and migration as well as increased procedural and fluoroscopic times, but the overall complication rate did not differ with TTE guidance when compared to historical controls that used TEE guidance.

## Introduction

Transesophageal echocardiography (TEE) and/or intracardiac echocardiography (ICE) are commonly used to guide transcatheter closure of secundum atrial septal defects (ASD) in clinical practice [1]–[5]. Our previous study has demonstrated that transcatheter ASD closure guided by TTE has a high success rate and a low complication incidence similar to TEE-guided procedures, and thus, TEE can be replaced by TTE in most patients undergoing transcatheter closure of ASD, especially in those with the central type of secundum ASD [6], [7]. However, the feasibility and safety of TTE-guided transcatheter closure of special types of secundum ASD remain unclear. Anatomically, a secundum ASD may extend into the superior sinus venosus (without superior-posterior rim), the inferior sinus venosus (without inferior-posterior rim), the mitral anaulus (without inferior-anterior rim) and the aortic root (without superior-anterior rim). It has been an international consensus that secundum ASD without superior-posterior or inferior-posterior or inferior-anterior rims should be closed surgically. However, it remains in dispute whether secundum ASD without superior-anterior rim can be closed with Amplatzer-type septal occluder (ASO). In the United States, the Amplatzer ASD occluder (St. Jude Medical, St. Paul, MN, USA) has not been approved for clinical use in patients with deficient ASD rims, though some studies have shown that transcatheter closure of secundum ASDs with deficient superior-anterior rims is safe and effective [8], [9], [10], [11]. Recently, transcatheter closure of ASDs with deficient superior-anterior rims by means of Amplatzer ASD occluder has been recommended as a relative indication by the 2009 Chinese Medical Association Expert Consensus, published in 2011 [12].

Nonetheless, special caution should be exercised in closing secundum ASD without superior-posterior rim because the ASO's rigid wire-right atrial disk interface may force the deployed left atrial disc to orient more or less perpendicular to the plane of the atrial septum, and the left atrial depth behind the aorta may be too short for the deployed left atrial disk, which may promote prolapse of the superior-anterior lip of the left atrial disc across the atrial septum to the right atrium. To ensure a successful closure of secundum ASD without superior-posterior rim, TEE is universally used in the literature to guide intervention, and it is unknown whether TTE can be used to replace TEE in this technically challenging situation. In this study, we retrospectively investigated the feasibility and safety of transcatheter closure of ASD with a deficient superior-anterior rim guided by TTE.

## Methods

### Study population

From January 2004 to June 2009, 325 patients with secundum ASD underwent transcatheter ASD closure in the Department of Cardiology and Department of Pediatrics, Qilu Hospital, Shandong University. Pediatric patients were excluded if their ages were less than three years and their total atrial septal length was less than the left disc diameter of ASO.

A total of 280 patients with a complete data set were enrolled in this study and divided into two groups. Patients in group A had a secundum ASD and a superior-anterior rim>4 mm (n = 118) and patients in group B had a secundum ASD and a superior-anterior rim ≤4 mm (n = 162). AS three cut-off values for a deficient superior-anterior rim (3 mm, 4 mm and 5 mm) existed in the literature [8], [9], [10], [11], we chose to use 4 mm as the cut-off value for a deficient superior-anterior rim of ASD.

Informed consent was obtained from the adult patients or the parents of the pediatric patients. The study protocol was approved by the Ethics Committee of Qilu Hospital, Shandong University.

### Echocardiographic evaluation and guidance

All adult and older pediatric patients (≥10 years) underwent local anesthesia, while pediatric patients younger than 10 years underwent general anesthesia.

The following Amplatzer-type septal occluders were used in the present study: Cardi-O-Fix ASD Occluder from Starway Medical Technology, Beijing, China, HeartR ASD Occluder from Lifetech Scientific, Shenzhen, China and MemoPart ASD Occlusion Device from Shanghai Shape Memory Alloy, Shanghai, China. All these occluders were self-expandable, double disc devices made from a nitinol wire mesh. The two discs are linked together by a short connecting waist corresponding to the size of the ASD.

**Figure 1:**
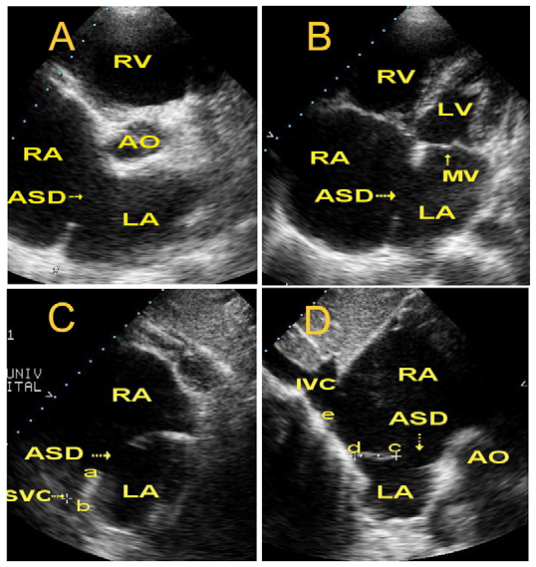
Measurement of ASD diameter and surrounding rims using TTE in different cross sections in a patient with ASD and a deficient superior-anterior rim. (A) Parasternal short-axis view showing measurement of the ASD diameter and the length of superior-posterior rim. The superior-anterior rim was absent. (B) Apical four chamber view showing measurement of the ASD diameter and the rim length from ASD to the mitral annulus and to the top of the atrium. (C and D) Subcostal view showing measurement of the ASD diameter, total atrial septal length, and rim length from the ASD to the mitral annulus and the superior vena cava (SVC) and inferior vena cava (IVC) entrances. Distance from “a” to “b” is the rim length of the SVC side, “c” to “d” rather than “c” to “e” is the rim length of the IVC side. The distance from “d” to “e” is the posteroinferior wall of the right atrium. ASD = atrial septal defect, AO = ascending aorta, LA = left atrium, RA = right atrium, LV = left ventricle, RV = right ventricle, IVC = inferior vena cava, SVC = superior vena cava.

Two ultrasound systems (HP Sonos 5500/7500 with an S4 transducer, Philips Healthcare, Eindhoven, The Netherlands, and GE Vivid 7 with an M3 S transducer, GE Healthcare, USA) with a central frequency of 2.0–4.0 MHz were used for two-dimensional (2D) echocardiographic and color flow mapping studies. Before intervention, all patients underwent TTE for determination of ASD number, size, position and spatial relations with adjacent cardiac structures. As a routine procedure, three cross sectional views were imaged for different measurements (Fig. 1). Parasternal short-axis view was first used to measure ASD diameter, the rim length from the ASD to the aortic root (superior-anterior rim), and the rim length from the ASD to the left atrial posterior and inferior wall (posterior-inferior rim). Then the apical four chamber view was imaged to measure the ASD diameter, the rim length from the ASD to the mitral annulus (anterior-inferior rim) and the rim length from the ASD to the top of the left atrium (posterior-superior rim). Finally, a serial of subcostal views were imaged to measure the ASD diameter, the total atrial septal length, the rim length from the ASD to the mitral annulus, the rim length from the ASD to the entrance of the superior vena cava (SVC), and the rim length from the ASD to the entrance of the inferior vena cava (IVC). As most atrial septal defects are oval-shaped, the largest ASD diameter may be detected in different cross sectional views in different patients, and thus the largest ASD diameter in a given patient was chosen as the reference ASD diameter for selecting the optimal size of ASO. In the majority of patients, the ASD rims in all orientations can be clearly visualized and measured directly by scanning multiple cross sections. However, in the minority of patients, the ASD rims in certain cross sectional views could not be clearly shown by 2D image and in these cases, the shunt flow displayed by color flow mapping and the ASD rims imaged in adjacent cross sectional views may help to estimate the rim length. In general, a deficient ASD rim (<7 mm near the mitral annulus or <5 mm near the SVC or IVC) constitutes contraindications [12] to transcatheter closure of ASD with ASO and these patients were intentionally excluded from this study.

During ASD closure, fluoroscopy was used to guide the catheter tip positioning and monitor ASO opening and closure, and TTE was used to guide ASO positioning and assess residual shunt. To ensure a proper ASO position in patients with a deficient superior-anterior rim, the following procedures were strictly followed.

#### Step one

From a right femoral vein approach, a long and stiff guidewire was inserted into the IVC, right atrium, ASD, left atrium and left upper pulmonary vein sequentially. Over the guidewire, an 8 Fr to 14 Fr sheath was passed into the left upper pulmonary vein. An ASO was attached to the delivery cable and pushed to the top of sheath. The left ASO disc was first opened in the left atrium, the sheath was pulled back gently to allow the left disc to press the atrial septum and ascending aorta tightly as certified by TTE, and then the waist and the right disc of the ASO were opened consecutively.

#### Step two

**Figure 2:**
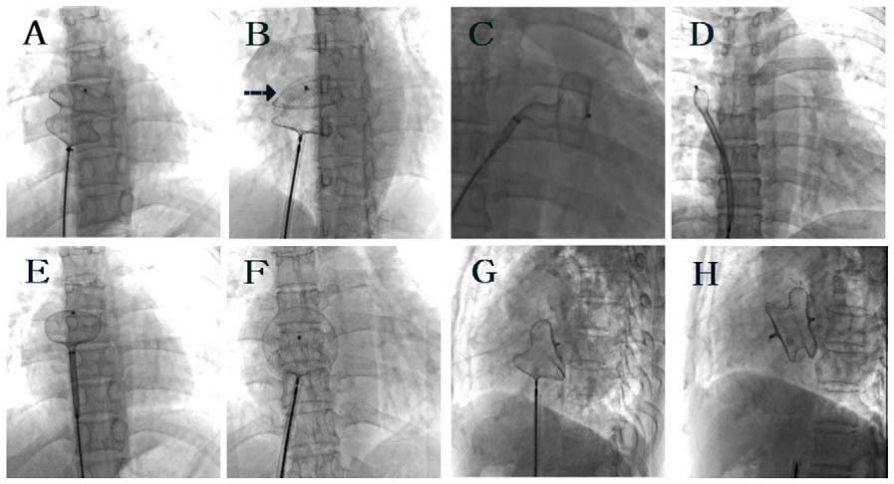
Fluoroscopy images of the Amplatzer-type septal occluder (ASO) in a patient with ASD and a deficient superior-anterior rim. (A and B) The two discs were seen separating clearly in the postero-anterior view, and the upper part of the left disc was shaped like a “beret” (arrow) in the left anterior oblique position, which demonstrated that the left disc upper part had already slipped into the right atrium. (C) The left disc was opened, and most of the waist was in the left atrium in an attempt to position the waist horizontally and to straighten the left disc. (D and E) The catheter sheath was introduced into right upper pulmonary vein, the catheter was pulled to the left atrium with the catheter orientation kept to the right upper side of the left atrium, and the whole left disc was open. (F and G) When the ASO was fully open at the ASD, the left and right discs were overlapped in the postero-anterior view and the upper part of the two discs were opened widely in a “Y” shape in the 45°–80° left anterior oblique view, the proper ASO position was assumed. (H) The ASO was released and the two discs were clearly separated.

**Figure 3:**
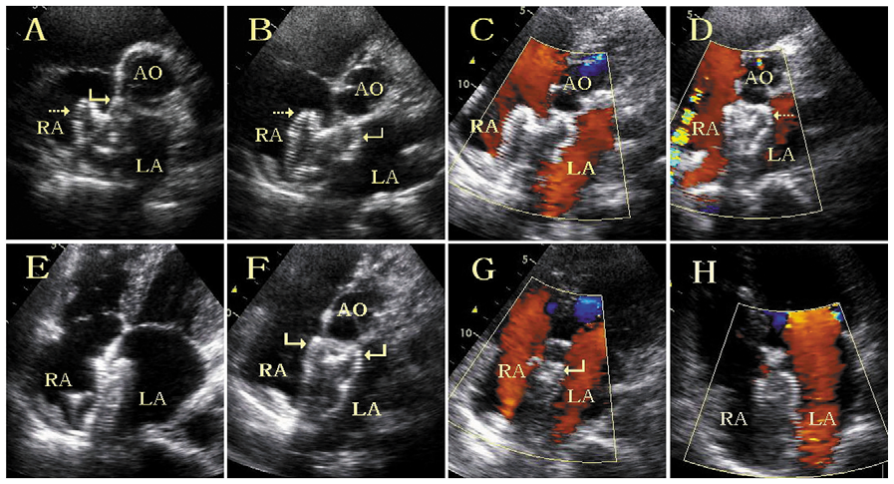
TTE images of the ASO in a patient with ASD with a deficient superior-anterior rim. (A) The left disc upper part was seen to be slipped into the right atrium (curved arrow). (B) After adjusted deployment, the upper parts of the two discs were positioned on the left and right side of the ascending aorta separately and opened as a “Y” shape (dashed and curved arrows). (C) No residual shunt was detected. The cable was pushed to make the right disc upper part press the ascending aorta tightly as shown in the parasternal short-axis view (D) and apical four-chamber view (E). After the ASO was released, the two ASO disc upper parts were positioned on the left and right side of the ascending aorta separately (curved arrows) (F) and no residual shunt was detected as shown in the parasternal short-axis view, the apical five-chamber view (G) and the apical four-chamber view (H). AO = ascending aorta, LA = left atrium, RA = right atrium.

Fluoroscopy was used to ascertain whether the left and right discs of ASO were positioned on the left and right side of the ascending aorta respectively (Fig. 2). A proper ASO position was assumed if the left and right discs almost overlapped in the postero-anterior position and the upper part of the two discs opened widely in a “Y” shape in the 45°–80° left anterior oblique position. On the other hand, if the two discs could be seen separating clearly in the postero-anterior position and the upper part of the left disc was shaped like a “beret” in the left anterior oblique position, the upper part of the left disc may already have slipped into the right atrium. Thereafter, TTE was used to further evaluate the ASO position (Fig. 3). When the ASO was opened completely, in the parasternal short-axis view, the two ASO disc upper parts were positioned on the left and right side of the ascending aorta separately and opened in a “Y” type, which signifies a satisfactory ASO position and shape. On the other hand, if the upper part of the left disc slipped into the right atrium and the upper part of both discs could be seen on the right atrium side of the ascending aorta, re-positioning of the ASO was required.

Despite these guidance procedures, it may be still difficult to position the two ASO discs on the left and right sides of the ascending aorta separately in some patients with a deficient superior-anterior rim, because the upper part of the left disc often sliped into the right atrium. To overcome this difficulty, apart from the method of opening the left disc in the left atrium as described in step one, we also used other two techniques. The first technique was to open the left disc and put most of the waist in the left atrium, and the catheter was then pushed upwards to position the waist horizontally and make the left disc downward. In this way, the orientation of the left disc will be changed from right upper-left lower to entirely vertical. Then, the catheter was gently pulled to the right to let the ASO waist pass through the ASD and let the left disc upper part press the left side of ascending aorta, and then the right disc was opened. Another method was to introduce the catheter sheath to the right upper pulmonary vein (Figure 2 D, E, F), partially open the ASO left disc in the right upper pulmonary vein, pull the catheter to the left atrium and keep the catheter orientation to the right upper side of the left atrium. The entire left disc was opened, the catheter was then pulled to let the left disc press the left side of the ascending aorta tightly, and the ASO waist and right disc were sequentially opened. In our experience, these two manipulating methods will greatly increase the success of the procedure.

#### Step three

When the ASO position was satisfactory, several echocardiographic views were used to confirm that the ASD rims were gripped tightly by the two discs, with no significant residual shunt flow detected. Under guidance of TTE and fluoroscopy, the cable was gently pushed and pulled to ensure that the occluder was well fixed. Once the foregoing criteria were satisfied, the occluder was finally released.

After the ASD closure procedure, TTE was performed immediately, 1 day, 1 month and 6 months in all patients to evaluate the effects and complications of transcatheter ASD closure. All heart chamber diameters were measured and pulmonary artery pressures were estimated in patients with tricuspid and/or pulmonary valve regurgitation. Residual shunt was assessed by color Doppler flow imaging [13]. The residual shunt was regarded as trivial if the width of the color jet was ≤1 mm, small if it was ≥1 mm and ≤2 mm, moderate if it was ≥2 mm and ≤4 mm and large if it was ≥4 mm. The procedure success was defined as no, trivial or small residual shunt immediately after the procedure [14]. Complications during and after transcatheter ASD closure were recorded.

### Statistical analysis

The Statistical Package for the Social Sciences v. 17.0 (SPSS, Inc., Chicago, IL, USA) was used for statistical analyses. Data were presented as the mean ± SD and range. Continuous variables were compared by Student's unpaired t test. Categorical variables were presented as numbers (%) and compared by Pearson's chi-squared test. A two-tailed *p*-value of <0.05 was considered statistically significant.

## Results

The baseline characteristics of the study population were shown in Table 1. There were no significant differences in age and gender between group A and group B. Group A and group B respectively contained 5 and 9 patients with pulmonary stenosis, 3 and 5 patients with atrial fibrillation, and 10 and 17 patients with interatrial septal aneurysms. In group A, one patient had a ventricular septal defect (VSD) and one had an acute myocardial infarction 20 days prior to admission. In group B, one patient had type II second-degree atrioventricular block and another had a patent ductus arteriosus (PDA).

**Table 1:** Baseline patient characteristics.

	Group A (superior-anterior rim>4 mm) (n = 118)	Group B (superior-anterior ≤4 mm) (n = 162)
Age (year, mean ± SD, range)	26.7±17.8 (3.0–74.0)	27.4±17.2 (3.0–67.0)
Male (n, %)	31 (26.3)	45 (27.8)
Pulmonary stenosis (n, %)	5 (4.2)	9 (5.6)
Atrial fibrillation (n, %)	3 (2.5)	5 (3.1)
Aneurysm of interatrial septum (n, %)	10 (8.5)	17 (10.5)
Other (n)	1 acute myocardial infarct1 ventricular septal defect	1 type II second-degree atrioventricular block1 patent ductus arteriosus

The results and complications of transcatheter ASD closure immediately after the procedure and at the 6-month follow-up visit were summarized in Table 2. In one patient from group A, cardiac catheterization confirmed anomalous inferior vena caval drainage into the top part of the right atrium, and ASO implantation failed in this patient. For 3 patients in group B, ASO implantation failed as the device was not able to be fixed in the proper position despite extensive efforts. ASO was successfully deployed in a total of 276 patients, 117 in group A and 159 in group B. The mean value of ASO-ASD in group B was significantly larger than that in group A. The mean procedure and fluoroscopy time in group B were much longer than those in group A. Combined PDA and VSD in 2 patients were closed successfully with TTE guidance. Fourteen patients with pulmonary stenosis underwent balloon valvuloplasty with a satisfactory result.

**Table 2:** Results and complications at the time of the procedure and at the follow-up visit.

	Group A (superior-anterior rim>4 mm, n = 117)	Group B (superior-anterior rim≤4 mm, n = 159)	*p* value
ASD diameter (mean ± SD, range, mm)	17.9±7.2(5.0∼33)	17.9±5.6 (8.0∼38)	0.994
ASO diameter(mean ± SD, range, mm)	23.0±8.7(8∼40)	24.9±7.0(12∼40)	0.130
ASO–ASD (mean ± SD, range, mm)	5.1±3.3 (0∼16)	7.0±2.7 (2∼14)	0.000
ASOs attempted (n)	1.14±0.43	1.19±0.46	0.391
1 ASO (n, %)	104 (88.9)	132 (83.0)	
2 ASOs (n, %)	11 (9.4)	23 (14.5)	
3 ASOs (n, %)	2 (1.7)	4 (2.5)	
Failure of ASO implantation (n, %)	1 (0.9)	3 (1.9)	0.428
Procedure time (min)	54.2±5.6	65.8±8.9	0.008
Fluoroscopic time (min)	12.6±3.8	16.9±5.7	0.016
Complications (n, %)	10 (8.5)	12 (7.5)	0.762
Transient hematuria	1	0	
Sinus bradycardia	2	0	
Type II second-degree AVB	3	2	
Migraine	1	2	
ASO migration	0	2	0.223
ASO malposition	1	4	0.307
Pericardial effusion	1	1	
Thrombogenesis on ASO	1	1	
Deaths	0	0	

ASD: atrial septal defect; ASO: Amplatzer-type septal occluder; AVB: atrioventricular block; ASO-ASD: the difference between ASO and ASD diameters.

Although the two groups did not differ in complications, ASO migration and malposition occurred more often in group B. In group B, two cases had ASO migration 9 and 12 hours after the procedure, requiring emergency cardiac surgery. Immediately after the procedure, ASO malposition, manifested as the anterior upper part of the left discs slipped into the right atrium, was detected by echocardiography in 4 cases of group B and 1 case of group A. One of these patients underwent cardiac surgery 48 days after the procedure because of continued palpitation and dyspnea, whereas another 3 patients had no serious symptoms. However, transcatheter closure of ASD in these 7 cases was performed in the early stage of ASO deployment in Qilu Hospital, Shandong University. Temporary complications after the procedure included 1 incident of transient hematuria, 2 incidents of sinus bradycardia, 5 of type II second-degree atrioventricular block, and 3 of migraine which resolved with treatment. Pericardial effusion was found in one patient from group A which disappeared after treatment, whereas a small amount of pericardial effusion combined with ASO malposition was detected in one patient of group B, which was persistent at follow-up. One day and 30 days after ASD closure, thrombus on the ASO surface was detected in 2 patients by TTE and disappeared 10 to 13 days after intensive anticoagulantion therapy. No aortic perforation or death occurred in both groups during procedure and at 6-month follow-up visit.

The success rate of ASD closure was summarized in Table 3. Group A and group B did not differ in the success rate of the procedure (98.3% vs. 95.0%) or complete closure rate immediately after the procedure (89.7% vs. 89.3%) and at 6-month follow-up visit (98.3% vs. 96.8%). Most of the 4 moderate residual shunts and the 1 large residual shunt in Group B were detected in patients with ASO malposition; these residual shunts were persistent at the 6-month follow-up visit.

**Table 3:** Immediate and follow-up ASD closure success rates.

	Immediate success	6-month success
Group A:		
Procedure success	116 (116/118, 98.3%)	
Complete closure	105 (105/117, 89.7%)	115 (115/117, 98.3%)
Trivial residual shunts	8	0
Small residual shunts	3	1
Moderate residual shunts	1	1
Large residual shunts	0	0
Group B:		
Procedure success	154 (154/162, 95.0%)	
Complete closure	142 (142/159, 89.3%)	151 (151/156, 96.8%)
Trivial residual shunts	7	0
Small residual shunts	5	2
Moderate residual shunts	4	3
Large residual shunts	1	0

ASD: atrial septal defect. Procedure success was defined as no, trivial, or small residual shunt immediately after the procedure, as assessed by color Doppler flow imaging. Groups A and B did not differ in complete closure either immediately after the procedure or at the 6-month follow-up visit.

## Discussion

The major finding of the present study was that in patients with secundum ASD who underwent TTE-guided transcatheter closure of ASD, the complication rate, procedure success rate and complete closure rate immediately after the procedure or at 6-month follow-up were similar in cases with a deficient or sufficient superior-anterior rim of the ASD. To the best of our knowledge, this study is the first to report TTE-guided transcatheter closure of ASD with a deficient superior-anterior rim, which appears feasible and safe.

Podnar et al. [15] examined the morphology of secundum ASD in 190 patients with TTE and TEE and found central defects in 46 patients (24.2%). Morphological variations were detected in 144 patients (75.8%), with the most frequent being deficiency of the superior-anterior rim in 80 patients (42.1%). After TEE examination, 151 patients underwent percutaneous closure with ASO, of these, 45 (29.8%) had central defects and 80 (53.0%) had defects with a deficient superior-anterior rim. Our experience supports their observation that ASD with a deficient superior-anterior rim is the most common morphological variation of the secundum ASD. This fact should be kept in mind by interventionists involved in congenital heart disease.

In the majority of previous studies of transcatheter closure of secundum ASD, TEE and/or ICE were commonly used for guiding ASO implantation, whether for central ASD or ASD with a deficient superior-anterior rim [8], [10], [11]. Although a few studies showed that TTE can be used for guiding or assisting the ASO implantation, a specific large-sample study of TTE-guided ASO implantation in patients with ASD and a deficient superior-anterior rim is lacking [6], [7], [9].

Huang et al. [8] investigated TEE-guided transcatheter closure of ASD with superior-anterior rim deficiency using ASO in 83 patients. There were 34 patients in group A (ASD with deficient superior-anterior rim) and 50 patients in group B (ASD with sufficient superior-anterior rim). The results showed that failure of ASO implantation occurred in 3 cases in each group. The immediate procedure success were 91.2% and 94%, respectively. Among 78 patients with successful implantation, 20 cases (25.6%) had small residual interatrial shunt on the next day and 5 cases had persistent small residual shunt during follow-up (6.4%).

In a series of 197 patients, Wang et al. [11] studied the short- and intermediate-term results of TEE-guided transcatheter closure of ASD with ASO. One hundred and fourteen patients (58%) with a deficient superior-anterior rim (<5 mm) were included as group A while 83 patients with an adequate superior-anterior rim comprised group B. Deployment of the ASO was successful in 191 patients and failed in 6. Repositioning of the device was required in 28 patients: 21 in group A and 7 in group B (21/114 vs. 7/83, P>0.05). The mean procedure time and fluoroscopic time were 86±13 min and 13.6±5.9 min, respectively. Three patients experienced severe complications: 1 had transient complete atrioventricular block, 1 tamponade requiring drainage, and 1 dislodgement of the device requiring emergent operation. A residual shunt was detected by TEE in 49 out of 191 patients (26%) 15 minutes after deployment: 34 in group A (31%) and 15 in group B (19%, *P*>0.05). In follow-up echocardiographic studies at 1 day, 3 months, 6 months, 12 months, and 24 months after device deployment, residual shunts were detected in 37/191 (19%), 15/189 (8%), 11/176 (6%), 7/131 (5%), and 3/72 (4%), respectively.

Another retrospective analysis of transcatheter ASD occlusion involved 69 patients [16]. The ASO was successfully implanted during 67 (97%) of 69 procedures. Deficient aortic rims were associated with more minor adverse events (p = 0.002) and reduced composite clinical success (p = 0.002).

The present study demonstrated that TTE-guided transcatheter closure of ASD with deficient superior-anterior rim is safe and reliable. The success rate of complete closure immediately after the procedure and at 6-month follow-up in group B with a deficient superior-anterior rim reached 89.3% and 96.8%, respectively. This result is comparable with those of transcather closure of ASD guided by TEE or ICE [8], [10], [11]. Nonetheless, among 280 patients undergoing transcatheter ASD closure, 4 cases had failed ASO deployment, 6 cases showed moderate or large shunt after the procedure, and 12 cases had procedure-related, albeit mild and temporary, complications. It should be noted that the failure of ASO deployment and ASO migration or malposition after intervention mainly occurred in the early phase of ASO deployment at our hospital, reflecting the learning curve of the procedure.

Procedure duration and fluoroscopic time are a major safety concern for both patients and physicians during transcatheter closure of ASD. In a previous study involving 200 patients, ASD closure was performed successfully under the guidance of TEE and the procedure duration and fluoroscopy time were 25–210 minutes (median 66 minutes) and 2.5–60 minutes (median 12 minutes), respectively [17]. The mean procedure duration and fluoroscopic time of Huang et al [8] and Wang et al [11] were previously mentioned. In the present study, the mean procedure time (54.2±5.6, 65.8±8.9 min) and fluoroscopic time (12.6±3.8, 16.9±5.7 min) guided by TTE were comparable to those in previous TEE-guided studies.

There are two important advantages of TTE over TEE in the guidance of transcatheter ASD closure. First, except for young children, general anesthesia was not required in most patients undergoing TTE-guided transcatheter ASD closure. As patients were conscious during the entire procedure, interventionists could communicate with their patients anytime and find out whether these patients may have any uncomfortable symptoms, which may represent serious problems such as pericardial effusion, hypotension, pulmonary embolism and angina pectoris, and once these complications occur, an emergency treatment can be promptly accomplished to prevent adverse outcome in these patients. Second, TEE is a semi-invasive method, which may pose the patient to the risks of laryngospasm, esophageal injury and perforation, aspiration pneumonia, arrhythmias and even death. The longer the procedure lasts, the more common the TEE-related complications would be. Thus, the closure procedure should be kept as short as possible when guided by TEE. By comparison, TTE is completely noninvasive and the interventionist can perform the closure procedure unhurriedly and thus a greater success rate may be achieved.

TTE is also advantageous over ICE in the guidance of transcatheter ASD closure in a number of ways. First, introduction of ASO catheter and ICE probe requires bilateral femoral vein puncture, which may cause complications both during and after the procedure. Second, manipulation of both ASO catheter and ICE probe in the right atrium may be technically difficult and sometimes induce complications. Third, ICE probe can be used only for once, which increases the economic burden on the part of the patient, particularly in the developing countries.

There are also two important disadvantages of TTE in the evaluation and monitoring of patients undergoing transcatheter ASD closure. First, in selecting optimal patients for ASO deployment, the inferior-posterior rim of ASD near the IVC may not be clearly seen in some patients as this rim can only be visualized in the subcostal views. Thus, TEE examination was required in these cases. Second, when the ASO was opened and positioned at the ASD, it was often difficult to determine whether the superior rim had been firmly clamped by the ASO discs and the following criteria were to be fulfilled before the ASD rim was considered to be clamped by the ASO discs: 1) The ASO was well fixed by a pull-and-push test; 2) The ASO discs could be seen separating clearly in the 45°–80° left anterior oblique view of fluoroscopy; 3) In parasternal short axis view, apical four-chamber and subcostal four-chamber views, the ASO shape and position were all satisfactory.

The major limitation of this study lied in its retrospective nature and the patients with ASD and a deficient superior-anterior rim were not randomized into transcatheter closure guided by TTE and TEE to compare the feasibility and safety of the two approaches. A large-sampled and randomized clinical trial is warranted for this purpose. Nevertheless, our approach provides a safe, simple and economical method to guide transcatheter ASD closure, which may play an important role in improving current clinical practice.

In conclusion, the absence of a sufficient superior-anterior rim in patients undergoing percutaneous closure of secundum-type ASDs using fluoroscopic and TTE guidance is associated with slightly greater device malposition and migration as well as increased procedural and fluoroscopic times. However, the overall complication rate did not differ with TTE guidance when compared to historical controls that used TEE guidance.
